# Genomic profiling of plastid DNA variation in the Mediterranean olive tree

**DOI:** 10.1186/1471-2229-11-80

**Published:** 2011-05-10

**Authors:** Guillaume Besnard, Pilar Hernández, Bouchaib Khadari, Gabriel Dorado, Vincent Savolainen

**Affiliations:** 1 Imperial College London, Silwood Park Campus, Buckhurst Road, Ascot SL5 7PY, UK; 2 CNRS, UPS, ENFA, Laboratoire Evolution & Diversité Biologique, UMR 5174, 31062 Toulouse 4, France; 3 Instituto de Agricultura Sostenible (IAS-CSIC), Alameda del Obispo s/n, 14080 Córdoba, Spain; 4 INRA, CBNMED, UMR 1334 Amélioration Génétique et Adaptation des Plantes (AGAP), 34398 Montpellier, France; 5 Dep. Bioquímica y Biología Molecular, Campus Rabanales C6-1-E17, Universidad de Córdoba, 14071 Córdoba, Spain; 6 Royal Botanic Gardens, Kew, Richmond TW9 3DS, UK

## Abstract

**Background:**

Characterisation of plastid genome (or cpDNA) polymorphisms is commonly used for phylogeographic, population genetic and forensic analyses in plants, but detecting cpDNA variation is sometimes challenging, limiting the applications of such an approach. In the present study, we screened cpDNA polymorphism in the olive tree (*Olea europaea *L.) by sequencing the complete plastid genome of trees with a distinct cpDNA lineage. Our objective was to develop new markers for a rapid genomic profiling (by Multiplex PCRs) of cpDNA haplotypes in the Mediterranean olive tree.

**Results:**

Eight complete cpDNA genomes of *Olea *were sequenced *de novo*. The nucleotide divergence between olive cpDNA lineages was low and not exceeding 0.07%. Based on these sequences, markers were developed for studying two single nucleotide substitutions and length polymorphism of 62 regions (with variable microsatellite motifs or other indels). They were then used to genotype the cpDNA variation in cultivated and wild Mediterranean olive trees (315 individuals). Forty polymorphic loci were detected on this sample, allowing the distinction of 22 haplotypes belonging to the three Mediterranean cpDNA lineages known as E1, E2 and E3. The discriminating power of cpDNA variation was particularly low for the cultivated olive tree with one predominating haplotype, but more diversity was detected in wild populations.

**Conclusions:**

We propose a method for a rapid characterisation of the Mediterranean olive germplasm. The low variation in the cultivated olive tree indicated that the utility of cpDNA variation for forensic analyses is limited to rare haplotypes. In contrast, the high cpDNA variation in wild populations demonstrated that our markers may be useful for phylogeographic and populations genetic studies in *O. europaea*.

## Background

In the last decades, major technical innovations have allowed a rapid development of various methods for genomic analysis. These have led to applications ranging from phylogeographical reconstructions to forensic analyses and species identification [1,2]. In plants, many studies have focused on the organelle genomes (i.e., plastid DNA - cpDNA - and mitochondrial DNA - mtDNA) for six major reasons: (*i*) these genomes are usually uniparentally inherited (either from the mother or the father) and thus allow for investigations of gene dispersal by seeds or pollen without recombination effect [3]; (*ii*) their haploid nature facilitates their sequencing and usually does not require cloning; (*iii*) such genomes are more prone to stochastic events because their effective population size is half that of diploid genomes, allowing a more accurate detection of evolutionary events such as a long persistence of relict populations in refuge zones during last glaciations [4]. In addition the dispersion of maternally inherited genomes (due to the seed dissemination only) occurs at shorter geographic distances than for nuclear genomes. The consequence of a reduced gene dispersal and high genetic drift in organelle genomes is a generally pronounced geographic structure, which facilitates phylogeographic analyses as well as tracing the origins of cultivated species or invasive populations [3]; (*iv*) they exhibit a high number of identical copies per cell [5], which may represent a significant advantage for forensic analyses; (*v*) they are circular and protected by a double-membrane envelope, which makes them resistant to exonucleases and less prone to endonuclease degradation (another advantage for forensics; [6]); and (*vi*) they exhibit a lower mutation rate than nuclear genomes [7,8], and such stability is generally required for traceability analyses (although see below).

The olive tree (*Olea europaea*, Oleaceae) is among the oldest woody crops, and nowadays represents one of the major cultivated species in the Mediterranean area [9]. The origins of this species have been recently investigated using different molecular techniques, including looking at organelle variation [10-15]. These previous studies allowed the detection of seven main cpDNA lineages in the *O. europaea *complex (for the olive tree classification see [16]): lineage E1 was detected in the Mediterranean area and Saharan Mountains, lineages E2 and E3 were specific to the Western Mediterranean area, lineage M was only detected in Macaronesia, lineages C1 and C2 were observed from Southern Asia to Eastern Africa, and lineage A was characteristic of Tropical African olives [15]. One limitation encountered during these studies was the particularly low level of cpDNA and mtDNA polymorphism in the Mediterranean olive tree. Until now only seven haplotypes have been detected with different combinations of loci [17,18]. These haplotypes belong to lineages E1, E2 and E3 (i.e., two or three haplotypes per lineage [15]). Recently, the first olive plastid genome (cpDNA) was released [18]. For detecting polymorphism in the cultivated olive tree, Mariotti and co-workers analysed sequence variation in 21 cpDNA fragments [18]. Variable microsatellites (also known as simple sequence repeats; SSR), insertions/deletions (indels) in repeated or non-repeated regions, and single nucleotide polymorphisms (SNPs) were identified and allowed for the identification of six cpDNA haplotypes (or chlorotypes) on a set of 30 cultivated olive trees, but they did not find new variants compared to previous studies [17]. The low cpDNA variation detected in the Mediterranean lineages hampered any applications of these markers, particularly for traceability or authenticity of olive oils [17]. Such a low level of cpDNA polymorphism has already been observed for other cultivated woody species such as *Prunus avium *[19], *Vitis vinifera *[20] and *Pinus pinea *[21]. This is probably due to human dispersal of cultivated genotypes originating from a reduced gene pool. In addition, low cpDNA polymorphism has also been reported in forest trees and this may also stem from low mutation rate in long-living organisms [22-24]. However, higher cpDNA variation has been detected in wild olives than in cultivars, and this allowed some population genetic analyses, for instance in the *laperrinei *and *guanchica *subspecies from Saharan Mountains and Canary Islands, respectively [25-27].

Additional investigations are needed to maximise the cpDNA haplotype identification in olive trees by testing new markers (especially multiallelic microsatellites [28]) on representatives of both cultivated and wild pools. Here, we address this challenge. Firstly, we sequenced the complete plastid genomes of seven *O. europaea *accessions, including one Spanish cultivar ('Manzanilla de Sevilla') and six wild olive trees. These taxa were chosen to represent the seven lineages previously reported in the olive tree complex [15]. We also report the complete plastid genome of *O. woodiana*, a taxon belonging to sect. *Ligustroides*, which is the sister clade to *O. europaea *[29]. Secondly, based on these genome sequences, we developed a method for a rapid and routine characterisation of length variation in 62 regions plus two cleaved amplified polymorphism sequence loci (CAPS). A set of 186 cultivars (including both major varieties and local types) as well as five distant wild olive tree populations (129 individuals) were characterised using this approach. Based on the observed polymorphism, we propose an optimised set of primers to detect Mediterranean haplotypes. We also discuss the utility of this approach for forensic analysis as well as for phylogeographic analyses of the olive tree complex.

## Results and Discussion

**Figure 1:**
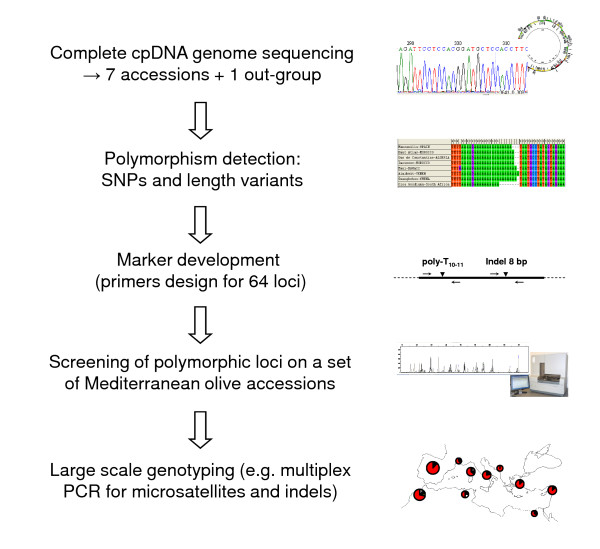
**Summary of our approach summary for developing a large-scale olive tree cpDNA genotyping method**.

In this study, eight complete olive tree plastid genomes were sequenced and deposited in GenBank/EMBL under the accession numbers FN650747, FN996943, FN996944, FN996972, FN997650, FN997651, FN998900 and FN998901. Polymorphisms were used for the development of new markers to scan cpDNA variation. These loci were used to characterise both cultivated and wild olive trees to assess their utility for forensic and phylogeographic studies. Our general approach is summarised in Figure 1.

### Variation in olive tree chloroplast genomes

The cpDNA genome sizes vary between 155,531 base pairs (bp; lineage C2; Almhiwit 5.1) and 155,896 bp (lineage M; Imouzzer S1). As suspected by Besnard & Bervillé [30] based on RFLPs, two long indels were observed in the seven olive tree cpDNA genomes: a 342-bp deletion (in the *ycf*1 gene) was observed in lineage E3 (Gué de Constantine 20), while a 225-bp deletion (in the *trn*Q*-rps*16 intergenic spacer) was detected in both individuals from South Asia (lineages C1 and C2). In addition, 15 smaller indels (i.e., inferior or equal to 12 bp, excluding microsatellite motifs) were also detected. Five of these indels correspond to the presence/absence of a repeated motif of seven to 12 bp (i.e., composed of one or two motifs; located at nucleotide 7,328, 9,526, 14,693, 83,196 and 85,059 in the 'Manzanilla de Sevilla' sequence; see GenBank/EMBL accession no FN996972).

Sequence variation was low, with a total of 218 substitutions on the seven olive plastid genomes. A maximum of 106 substitutions (0.07%) was detected between Gué de Constantine 20 (Algeria) and Almhiwit 5.1 (Yemen), while cpDNA genomes of Guangzhou 1 (China) and Almhiwit 5.1 (Yemen) only showed 34 substitutions (Additional file 1). The plastid genome of *O. woodiana *displays between 417 and 432 substitutions (< 0.28%) when compared to the seven *O. europaea *genomes. Again, this level of variation is surprisingly low if we consider that the divergence between sections *Olea *(*O. europaea*) and *Ligustroides *(*O. woodiana*) is estimated to be between 14 and 22 million years (My; [29]). Based on these results, the cpDNA substitution rate was estimated to be between 1.2 × 10^-10 ^and 2 × 10^-10 ^in the *Olea *subgenus, which is about ten times lower than the typical mutation rate reported for the plastid genome [7]. This slow molecular evolution might be related to the long generation time of the olive tree [23,24].

Twelve differences (i.e., three length polymorphisms and nine SNPs, of which one is located in the inverted repeat) were observed between the genomes of 'Frantoio' (GenBank/EMBL accession GU931818; Italy; [18]) and 'Manzanilla de Sevilla' (Spain; this study). According to our approach, we re-sequenced the variable regions in 'Frantoio', from the Olive World Germplasm Bank (OWGB) at Córdoba, Spain (GenBank/EMBL accessions no. FR754486 to FR754495), but these polymorphisms were not confirmed. These 12 differences are not located in the cpDNA regions screened for sequence variation by Mariotti et al. [18] and may be seen as putative sequencing mistakes in accession GU931818. Considering this fact, our analyses indicate that 'Frantoio' and 'Manzanilla de Sevilla' display the same plastid genome, supporting a common maternal origin for these two cultivars.

**Figure 2:**
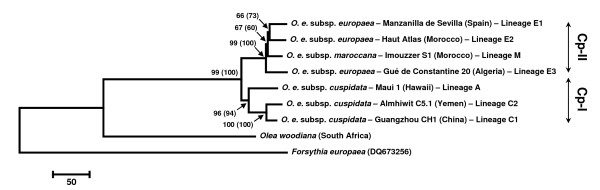
**Plastid DNA phylogenetic tree of the seven olive tree lineages based on nucleotide substitutions from complete plastid genomes**. The same topology was obtained with maximum parsimony and maximum likelihood (GTR+I+G) analyses. The bootstrap values are given on each branch (when superior to 50%), the first corresponding to the MP analysis and the second (in brackets) to the ML analysis. The *Forsythia europaea *and *Olea woodiana *sequences were used as outgroups. The tree was rooted with the *Forsythia *sequence. The two clades Cp-I and Cp-II are indicated according to Besnard et al. [15].

Based only on nucleotide substitutions (i.e., only 65 out of 218 substitutions were parsimony-informative in the olive tree complex), phylogenetic relationships were depicted from the complete cpDNA genomes using both maximum parsimony (MP) and maximum likelihood (ML) techniques (Figure 2). The resulting topologies confirm results from Besnard et al. [15,29] through the recovery of two main clades: a Mediterranean/North African clade (clade Cp-II) including lineages E1, E2, E3 and M, and a *cuspidata *clade (clade Cp-I) including lineages C1, C2 and A. In clade Cp-II, moderate bootstrap support for an early-diverging position of lineage E3 (Gué de Constantine 20) agrees with results based on a few cpDNA microsatellites, indels and CAPS [15]. A moderate level of support was also recovered for the clustering of lineages E1 and E2. Only nine informative substitutions were detected in clade Cp-II, three of them being non-synonymous (Table 1). The information brought by these sites does not strongly support any relationship, suggesting that some sites may be homoplastic. Indeed, two of the three non-synonymous substitutions (52,165 and 83,304) are polymorphic in both clades Cp-I and Cp-II, suggesting that these sites could be under selective pressures, either maintaining polymorphism or contributing to the recurrent appearance of the same substitution (see also [18]). Understanding the molecular variation at these non-synonymous sites would deserve the design of an experiment to test their origin and their adaptive significance.

**Table 1:** Nucleotide polymorphisms at the nine parsimony informative sites for clade Cp-II (lineages E1, E2, E3 and M).

	Sites ^a^								
	
Accession	9,081	31,283	48,091	51,579 (*psb*G)	52,165 (*ndh*C)	67,653	83,304 (*rpl*14)	112,753 (*ndh*F)	122,532
*O. woodiana*	C	T	C	A	T	T	G	A	G
									
Maui 1	C	T	C	A	**G**	T	G	**C**	G
Almhiwit 5.1	C	**G**	C	A	T	T	G	**C**	G
Guangzhou 1	C	T	C	A	T	T	**T**	**C**	G
									
Gué de Constantine 20	**T**	T	**A**	A	T	T	G	**C**	G
Imouzzer S1	C	T	**A**	**C**	T	**G**	G	**C**	**T**
Haut Atlas	**T**	T	**A**	**C**	T	**G**	**T**	A	G
Manzanilla de Sevilla	**T**	**G**	C	**C**	**G**	**G**	**T**	A	**T**

					*		*	*	
Non-synonymous sites					L/F		F/L	L/W	

^a ^Sites are defined by their location in the 'Manzanilla de Sevilla' sequence. When the site is located in a coding sequence, the gene name is given in brackets.

* For non-synonymous substitutions, amino-acid changes are indicated below.

### Development of cpDNA markers

The low cpDNA substitution rate combined with possible selective effects (which can be problematic for phylogenetic reconstructions [31]) led us to focus on "length polymorphisms". Such polymorphisms were either the result of a variable number of repeats in a microsatellite motif (referred as "microsatellites"), or another type of insertion/deletion (referred as "indel"). Sixty-two regions, of which 51 display variable microsatellite motifs, were investigated (Additional file 2). These sites are located in non-coding regions (except for loci 61 in *ycf*1) and can thus be considered as mostly neutral. The list of polymerase chain reaction (PCR) primers to amplify the 62 regions is given in Additional file 2. Two CAPS loci (located in *rpl*14 and the *petA-psbJ *intergenic spacer) were also characterised to allow the distinction of new haplotypes in lineage E1 (see Methods). After the characterisation of 315 cultivated and wild trees, a multilocus profile (or cpDNA haplotype) was defined for each individual (Additional file 3a). Also, an 88-year old herbarium leaf sample was successfully characterised, suggesting that our method is appropriate for investigating cpDNA variation even on poorly preserved DNA. A total of 40 loci were polymorphic in the Mediterranean/North African olive tree (Additional file 3b). We hope that data generated using this method by different laboratories could be compared to generate a reference dataset for the Mediterranean olive tree. In this way, it should be possible to reconstruct a detailed phylogeography of the species based on a large number of populations, as has been done, for instance, for the European white oaks [32].

### Polymorphism assessment in the Mediterranean olive

**Figure 3:**
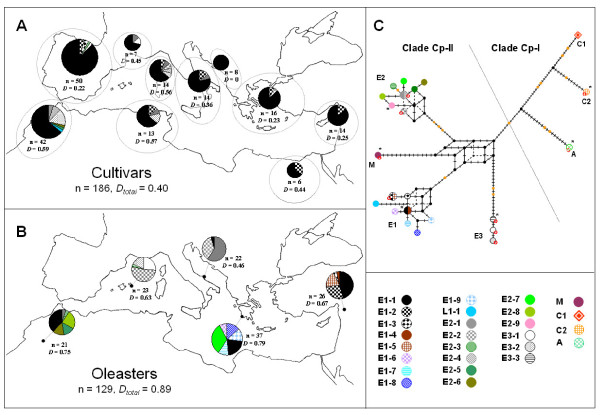
**Plastid DNA variation in the Mediterranean olive trees**. **A**. Distribution of the cpDNA haplotypes in cultivated olive trees (see also Additional file 5 for the list of cultivars and the corresponding cpDNA haplotype). **B**. Distribution of haplotypes in the five studied oleaster populations. For both cultivated and wild gene pools, the number of accessions (n) and the discriminating power (*D*, *D_total_*) of cpDNA variation is given for each region or population and on the global sample. **C**. Reduced-median network [54] of cpDNA haplotypes. The traits on branches represent each individual change. Indels are specifically distinguished by bigger orange traits. Each haplotype is represented by a symbol with a definite colour. The name of each cpDNA clade or lineage is given according to Besnard et al. [15] (see also Figure 2). The missing, intermediate nodes are indicated by small black points. CAPS-*Xap*I and CAPS-*Eco*RI were not considered in this analysis. For this reason, three pairs of haplotypes (i.e., E1-1/E1-4, E1-2/E1-5 and E2-1/E2-4) are not distinguished in the network. In addition, the nine haplotypes not restricted with *Xap*I are indicated with a red circle. * haplotypes for which a complete genome was released in the present study.

Some olive tree varieties are used to produce high-quality (and thus more expensive) extra virgin olive oil. Therefore, they may be granted a label of protected designation of origin (PDO; a European Union label referring to food products specific to a particular region or town, conveying a particular quality or characteristic of the specified area). Our markers could find some applications in the traceability of such high quality olive oils, but their discriminating power needs to be determined for assessing their putative utility. Using our cpDNA loci, 12 haplotypes were detected in cultivars (Table 2, Figure 3a and Additional file 3): hence our approach permitted a two-fold increase of the number of detected variants compared to previous studies [17,18]. The most frequent haplotype (E1.1) was detected in 77% of cultivars, including 'Frantoio' and 'Manzanilla de Sevilla'. Two other haplotypes (E1.2 and E3.2) displayed a frequency superior to 5%, but the remaining haplotypes were rare, and sometimes detected only once (i.e., L1.1, E2.3, E2.5 and E2.6) or twice (i.e., E1.3, E2.2 and E3.1). Several of these rare haplotypes were detected in local cultivars with a limited economic importance (e.g., E2.5, E2.6 and L1.1). The probability that two samples chosen at random display a different haplotype was low (*D *= 0.40) when compared to nuclear markers, especially nuclear microsatellites for which the discriminating power per locus generally exceeds 0.70 [33-35]. This indicates that the utility of the cpDNA variation for forensic analysis is restricted to rare haplotypes such as the ones detected for 'Picholine' (E2.1) and 'Olivière' (E3.1) in France, 'Villalonga'-'Blanqueta' (E1.3), 'Farga' (E3.1) and 'Lechín de Sevilla' (E2.3) in Spain, or 'Megaritiki' (E2.2) in Greece. These varieties are used to produce high quality extra virgin olive oil (e.g., for Spanish cultivars see [36]). The cpDNA variation, which is *a priori *easily analysable compared to nuclear single-copy genes, should thus be helpful to complement other procedures for olive traceability based on nuclear polymorphisms [e.g., [37]].

**Table 2:** Frequency of each haplotype in cultivars (186 individuals) and oleaster populations.

	Haplotype frequency (%)					
	
Haplotype *	Cultivars	Bin El Ouidane	Minorca	Pugnochiuso	Gialova	Rajo
						
E1.1	77.0	42.9	-	4.5	21.6	46.2
E1.2	7.0	-	-	-	-	26.9
E1.3	1.1	-	-	-	-	3.8
E1.4	-	-	-	-	-	19.2
E1.5	-	-	-	-	-	3.8
E1.6	-	-	-	-	8.1	-
E1.7	-	-	-	-	10.8	-
E1.8	-	-	-	-	13.5	-
E1.9	-	-	-	-	13.5	-
L1.1	0.5	-	-	-	-	-
						
E2.1	3.2	4.8	-	68.2	-	-
E2.2	1.1	-	52.2	27.3	-	-
E2.3	0.5	4.8	4.3	-	-	-
E2.4	2.1	-	-	-	-	-
E2.5	0.5	14.3	-	-	-	-
E2.6	0.5	23.8	-	-	-	-
E2.7	-	-	-	-	32.4	-
E2.8	-	14.3	-	-	-	-
						
E3.1	1.1	-	26.1	-	-	-
E3.2	5.3	-	17.4	-	-	-

* See Additional file 2 for the haplotype profile definition.

In the five populations of oleasters, 18 cpDNA haplotypes were detected, ten of which were shared with cultivars (Table 2, Figure 3b and Additional file 3). The discriminating power of cpDNA was high in these populations (*D *= 0.89) compared to the cultivated olive tree. Fourteen haplotypes were unique to one population, while the four remaining haplotypes were shared between at least two populations: E1.1 (Rajo, Gialova, Pugnochiuso and Bin El Ouidane), E2.1 and E2.2 (Bin El Ouidane and Pugnochiuso) and E2.3 (Minorca and Bin El Ouidane). These four haplotypes have been detected in cultivated olive trees and could reflect long-distance gene flow mediated by humans [15,38]. In this way, the most frequent haplotype in cultivars (E1.1) is also the most frequent and widespread haplotype in oleasters (22%; Figure 3b).

### Implications for phylogeography

Previous cpDNA phylogeographic studies of the Mediterranean olive tree have been limited due to the low number of haplotypes detected [17,18]. Here, we demonstrate that a genomic profiling approach of the plastid DNA mostly based on microsatellites and indels can solve this problem. The high variation detected in five distant wild populations indicates a high potential of our approach for resolving the Mediterranean olive tree history. One putative limitation is the level of homoplasy on microsatellite motifs, reported by different authors [39-42], and which could prove problematic when accurately identifying evolutionary relationships between haplotypes. We reconstructed a reduced median network based on molecular markers (Figure 3c). The Mediterranean haplotypes clustered into three lineages (E1, E2 and E3), while the haplotype of subsp. *maroccana *formed a fourth lineage (M) in northern Africa. This topology is fully congruent with Besnard et al. [15,29], who used different cpDNA data (i.e., microsatellites, indels and CAPS, or nucleotides). Each lineage displays at least one specific indel, with the exception of lineage M (Figure 3c). Phylogenetic relationships remain unresolved at the base of lineages E1 and E2, as well as in the centre of the network, as a consequence of homoplasy between haplotypes belonging to different lineages (e.g., shared length polymorphisms between clades Cp-I and Cp-II at loci 1, 2, 9, 17, 25, 38, 47, 48, 49, 50 and 58; Additional file 3). Such a difficulty for determining the ancestral state hampers the correct identification of historical links between divergent lineages. In contrast, we expect that homoplasy will not be a serious limitation to resolve phylogenetic relationships among lineages, since their haplotypes have diverged more recently [42]. In any case, for an optimal analysis of the cpDNA variation at the population level, possible length homoplasy will need to be considered and the use of appropriate models will be necessary [41,43].

The partial or complete cpDNA sequencing of new individuals may reveal nucleotide substitutions that would be of interest [18] for the development of new molecular markers like SNPs (or CAPS). Such SNPs could be used to improve our approach. Nevertheless, the homoplasy is not restricted to repetitive sequences as illustrated with non-synonymous sites in genes under selection, such as the polymorphism detected at the CAPS-*Xap*I locus (in *rpl*14; Table 1). In the present study, we found restriction polymorphism at this locus in lineages E1 and E2 (clade Cp-II) and also in clade Cp-I (for which we analysed only three accessions; Figure 3c) indicating that this site is highly homoplastic (see also Mariotti et al. [18]). Thus, this site should be used with caution for phylogeographic purposes. Nevertheless, we consider that it could bring potentially important information at the lineage level, particularly to solve the origin of haplotype E1.2 in the cultivated gene pool (7% of cultivars).

## Conclusions

A set of 40 polymorphic loci (including 35 with microsatellite motifs) is released for a rapid cpDNA characterization of the Mediterranean olive tree germplasm (see Methods, and Table 3). We expect that, besides their potential forensics application, their use will be important for phylogeographic analyses. Particularly, such studies should allow testing for the persistence of relict populations in the Mediterranean Basin [44], as well as to test the hypotheses about their post-glacial expansion and subsequent domestication [15,45]. In addition, the identification of genuinely wild populations may represent a significant evolutionary heritage for the conservation of the Mediterranean olive tree diversity. Lastly, the combined use of both nuclear and cpDNA resources should be useful to disentangle the impact of gene dispersal by seeds and pollen on the structure of the genetic diversity. For example, our cpDNA markers will have applications for a comparative study of the dynamic of wild olive tree populations in different environments, such as archipelagos and Saharan mountains [25,26]. Such information may be relevant for defining appropriate strategies of prospection and *in situ *conservation of the wild olive tree.

**Table 3:** Multiplexes of polymorphic loci (with their allele size range in bp) for characterizing the Mediterranean olive tree germplasm *.

**Multiplex PCR**	**Locus no**.	**Allele size range (bp)**	**Multiplex PCR**	**Locus no**.	**Allele size range (bp)**
	
A-1 (NED-M13)	46	110-112	B-2 (HEX-M13)	48	158-159
	1	121-124		25	174-177
	9	135-136		36	182-183
	51	139-146		52	191-203
	22	158-159		58	234-236
	41	169-171			
			C-1 (FAM-M13)	21	103-104
A-2 (NED-M13)	17	178-179		38	109-111
	28	182-183		31	131-133
	56	188-190		15	137-138
	53	203-204		47	154-157
	50	227-228		59	164-165
	33	235-236			
			C-2 (FAM-M13)	6	173-174
B-1 (HEX-M13)	39	105-106		49	181-182
	27	112-113		24	187-189
	23	120-121		29	203-204
	11	126-136		57	224-227
	42	137-139		54	231-239
	2	148-150			

* After PCR, the six multiplex PCRs (35 loci) were mixed together with locus 10 (allele size range of 87 to 95 bp) and ROX 500 as internal standard, and then run on an ABI Prism 3100 Genetic Analyzer.

## Methods

The general approach is summarised in Figure 1.

### Chloroplast genome sequencing

In order to maximize polymorphism detection, the analysis focused on seven individuals of *O. europaea *L. (subgenus *Olea *sect. *Olea*, or olive tree complex), which were chosen to represent one haplotype of each previously described lineage [15]. The following genotypes were thus investigated: 'Manzanilla de Sevilla' (Spanish cultivar; lineage E1), oleaster "Haut Atlas 1" (Morocco; lineage E2), oleaster "Gué de Constantine 20" (Algeria; lineage E3), subsp. *maroccana *"Imouzzer S1" (Morocco; lineage M), subsp. *cuspidata *"Maui 1" (Hawaii; lineage A), subsp. *cuspidata *"Guangzhou CH1" (China; lineage C1), and subsp. *cuspidata *"Almhiwit C5.1" (Yemen; lineage C2). In addition, we characterised one outgroup species [*O. woodiana *Knobl. subsp. *woodiana *(South Africa); sect. *Ligustroides *Benth. & Hook.], which belongs to the sister group of *O. europaea *[16,29]. Appropriate PCR primers were designed to amplify 105 overlapping cpDNA fragments (Additional file 4). Each PCR reaction (25 μl) contained 10 ng DNA template, 1× reaction buffer, 2 mM MgCl_2_, 0.2 mM dNTPs, 0.2 μmol of each primer, and 0.75 U of *Taq *DNA polymerase (Promega, Madison, WI, USA). The reaction mixtures were incubated in a thermocycler (T1; Biometra, Göttingen, Germany) for 2 min at 95°C, followed by 36 cycles of 30 s at 95°C (denaturing), 30 s at the annealing temperature (Additional file 4), and 2 min at 72°C (extension). The last cycle was followed by a 10-min extension at 72°C. Direct sequencing of PCR amplicons was performed with an ABI Prism 3100*xl *Genetic Analyzer, using the Big Dye v3.1 Terminator cycle-sequencing kit, according to the manufacturer's instructions (Applied Biosystems, Foster City, CA, USA). Additionally, nested (internal) primers were also designed to complete the sequencing of each fragment (Additional file 4). The eight *Olea *genomes were thus reconstructed using a similar approach to the one used by Mariotti et al. [18].

### Characterisation of cpDNA polymorphisms in the Mediterranean olive tree

Based on the seven *O. europaea *sequences, length polymorphism was detected in 62 regions. These polymorphisms were either due to a variable number of repeats in a microsatellite motif or another type of indel (Additional file 2). The PCR primers were designed in flanking regions to specifically amplify short segments (generally inferior to 240 bp). For locus multiplexing, the annealing temperature of all these primers needed to be similar, while the size of PCR products of each locus should be as different as possible. Finally, these primers were also designed to allow amplification of short DNA segments for characterization of poorly preserved material and highly degraded DNAs from herbarium samples. Additionally, the 5' end of the reverse primer of locus 19 was tagged with the sequence GTGTCTT to minimize band stuttering. All primer pairs and specific characteristics of generated fragments are given in Additional file 2. To reduce the cost of the PCR characterization (i.e., time and costs), we used the method described by Schuelke [46]. For each locus (except loci 8, 10, and 61), an 18-bp tail of M13 was added on the forward primer (Additional file 2). When each locus was amplified separately, each PCR reaction (25 μl) contained 10 ng DNA template, 1× reaction buffer, 2.5 mM MgCl_2_, 0.2 mM dNTPs, 0.2 μmol of one universal fluorescent-labelled M13(-21) primer (5'-TGTAAAACGACGGCCAGT-3'; labelled with one of the three following fluorochromes: HEX, 6-FAM or NED), 0.2 μmol of the reverse primer, 0.05 μmol of the forward primer, and 0.5 U of *Taq *DNA polymerase (Promega). The reaction mixtures were incubated in a T1 thermocycler for 2 min at 95°C, followed by 28 cycles of 30 s at 95°C, 30 s at 57°C, and 1 min at 72°C, and then by 8 cycles of 30 s at 95°C, 30 s at 51.5°C, and 1 min at 72°C. The last cycle was followed by a 20-min extension at 72°C. Usually, we amplified five or six loci in the same reaction, but in this case, the MgCl_2 _concentration was increased to 5 mM, and the concentration of primers (except the labelled M13 primer) was decreased by five or six. Loci 8, 10, and 61 (without the M13 tail) were amplified separately with the following conditions: each PCR reaction (25 μl) contained 10 ng DNA template, 1× reaction buffer, 2 mM MgCl_2_, 0.2 mM dNTPs, 0.2 μmol of each primer, and 0.75 U of *Taq *DNA polymerase. The reaction mixtures were incubated in a T1 thermocycler for 2 min at 95°C, followed by 36 cycles of 30 s at 95°C, 30 s at 53°C, and 2 min at 72°C. The last cycle was followed by a 10-min extension at 72°C.

The PCR products labelled with a fluorochrome were mixed together with GeneScan-500 ROX as internal standard to run the maximum of loci at the same time (considering the colour and the expected allele size range). They were separated on an ABI Prism 3100*xl *Genetic Analyzer and the fragment size was determined with GeneMapper version 4.0. For the two non-labelled loci 8 and 61, indels of 342 and 225 bp were revealed under UV after migration on a 2.5% agarose gel electrophoresis stained with GelRed (Biotium, Hayward, CA, USA).

We also focused on the characterisation of two substitutions, which were detected by Mariotti et al. [18] in lineage E1 (the most frequent one in cultivated olive trees; see [13,17]) and may be potentially useful for forensic analyses and the study of olive tree domestication. We chose to develop two Cleaved Amplified Polymorphism Site (CAPS) loci as in Besnard et al. [47], in order to rapidly characterise a high number of individuals. The PCR primers are given in Additional file 2. The two loci were amplified following the same PCR conditions as for microsatellites. The PCR products were digested with a restriction enzyme (*EcoR*I or *Xap*I) according to the manufacturer recommendations. The restricted fragments of the two loci were then mixed (with the internal standard ROX 500) and separated on an ABI Prism 3100 *xl *Genetic Analyzer. The polymorphism for the presence/absence of a restriction site was scored for each genotype. The possibility of multiplexing three different colours (e.g., NED, FAM and HEX) allows the characterisation of 288 (96 × 3) samples per run.

We then characterised 186 cultivated olive tree accessions from different areas with the 64 loci (Table 2, Figure 3a and Additional file 5). The analyzed germplasm includes 106 cultivars from the OWGB Córdoba [48]. These cultivars represent major cultivars from all Mediterranean countries. A few local cultivars from different places were also included in our study for a better representativeness of the cultivated gene pool. First, we characterized 55 cultivated local forms from Morocco (41) and Corsica-Sardinia (14) previously genotyped with nuclear markers [49,50]. In addition, cultivated trees with or without known denominations from Algeria-Tunisia (6), Italy (6), France (2), Greece-Turkey (3), the Levantine region (5), Libya-Egypt-Sudan (2) and South Africa (1) were added to this study. Beforehand, we tested with nuclear microsatellites that these latter accessions were genetically different (G. Besnard, unpubl. data), except for one herbarium leaf sample from Kufra, Libya (Newberry, sn; 1933 - Kew Herbarium). In addition, to assess the cpDNA variation in the wild Mediterranean olive trees, 129 individuals from five distant populations (Figure 3b) were also characterized: Rajo (Syria; 36°43'50''N, 36°40'00''E), Gialova (Greece; 36°55'12''N, 21°42'42''E), Pugnochiuso (Italy; 41°47'46''N, 16°10'05''E), Minorca (Spain; 39°56'52''N, 04°14'42''E) and Bin El Ouidane (Morocco; 32°03'00''N, 06°35'00''W). To test the reproducibility of the method, the characterisation of ten accessions (i.e., 'Picholine Marocaine', 'Manzanilla de Sevilla', 'Frantoio', 'Moraiolo', 'Ciarasina', 'Confetto', 'Itrana', 'Giaraffa', 'Kalamon' and 'Souri') were repeated three times at random.

Based on this analysis of wild and cultivated accessions, 40 polymorphic loci were detected in the Mediterranean olive trees (Additional file 3). We first proposed to combine 36 of these loci for a rapid characterisation of Mediterranean olive tree germplasm. The multiplex PCRs of five or six loci are proposed in Table 3, but this can be easily modified. The PCR conditions are those previously reported (with the M13 primer). After PCR, these products are mixed together (with no overlap for allele size between loci in a given colour). The locus 10, which needs to be amplified separately, is combined with these multiplex PCRs. Second, when amplified in a multiplex PCR, we encountered some difficulties with locus 19 (not reported in Table 3), and we thus recommend to use it separately and to combine it with the two CAPS (CAPS-*Xap*I and CAPS-*Eco*RI) for a second combination of three loci. Lastly, the locus 61 is independently characterised on 2.5% agarose gel electrophoresis.

### Data analysis

A phylogenetic tree based on the complete plastid genomes was constructed. A partial cpDNA sequence of *Forsythia *(DQ673256; [51]) was used as an outgroup to root the tree. Sequences were aligned with the application MEGA v4.1 [52]. The alignment was manually refined. Firstly, a maximum parsimony analysis was performed. All characters were equally weighted. The gaps were treated as missing data. A heuristic search was used to find the most parsimonious trees. The close-neighbor-interchange algorithm was used with a search level of 3, as recommended and implemented in the software [52]. The searches included 100 replications of random addition sequences. All the best trees were retained. A strict consensus tree was generated from the equally most-parsimonious trees. The bootstrap values were computed using 10,000 replicates. Secondly, the tree inference was made under a maximum likelihood criterion, using the application PHYML v3.0 [53]. The best-fit substitution model, determined through hierarchical likelihood ratio tests, was the GTR model, with invariable sites and a gamma shape parameter estimated from the data. Support values were obtained by 1,000 bootstrap replicates. Based on fragment genotyping (i.e., microsatellites and indels), the relationships among cpDNA haplotypes were visualized by constructing a reduced median network implemented in the application NETWORK v4.112 [54]. Multi-state microsatellites were treated as ordered alleles and coded by the number of repeated motifs for each allele (e.g., number of T or A; see also [15]) whereas the presence or absence of other indels was coded as 1 and 0, respectively. Basically, this coding strategy assumes that variation at cpDNA microsatellites is mainly due to single-step mutations (e.g., [15,18]), while allowing consideration of length polymorphisms (microsatellites or indels) with similar weight. However, whether we used different weights or not for indels versus microsatellites did not affect the topology. In addition, for loci combining indels and microsatellite motifs (loci 10, 11, 54 and 57), we separately coded the two types of characters based on available sequences for these loci. The matrix used for the analysis is given in Additional file 6.

The probability that two individuals taken at random display a different haplotype was computed as *D *= 1 - Σ *p_i_*^2^, where *p_i _*is the frequency of the haplotype *i*. This parameter was calculated separately on cultivated and wild olive trees, but also on sub-samples or populations. The groups of cultivated olive trees were defined according to their geographic origin.

## Authors' contributions

GB & VS designed the initial project, with subsequent contributions by the other authors. GB conducted the experiments and wrote the initial version of the manuscript. GD and PH contributed to olive cpDNA sequencing and to the acquisition of cultivated olive genotyping data. BK contributed to the acquisition of data for cultivated and wild olive trees. GB, PH, BK, GD and VS revised the manuscript critically. All authors have given final approval for this version to be published.

## Supplementary Material

Additional file 1 **Nucleotide substitutions between each pair of *Olea *plastid genomes**. Click here for file

Additional file 2 **Loci features**. Primers, allele size range, polymorphism type, genome location and corresponding names in previous studies are given Click here for file

Additional file 3 **Plastid DNA variation based on the 64 loci**. **a) **Profiles for the 321 trees characterized in this study (including those for complete cpDNA genomes); and **b) **Different cpDNA haplotypes. Click here for file

Additional file 4 **PCR amplification and sequencing primers (5'->3') used to amplify and sequence the complete olive plastid genome**. Click here for file

Additional file 5 **Characterised cultivars and their cpDNA haplotypes**. Click here for file

Additional file 6 **Data matrix of the 26 cpDNA haplotypes for the reduced-median network analysis**. Click here for file
