# Supplementary material for: Genomic profiling of plastid DNA variation in the Mediterranean olive tree
Source: BMC Plant Biol. 2011 May 10;11:80. doi: 10.1186/1471-2229-11-80 (PMC3115843; doi:10.1186/1471-2229-11-80)
Supplement: Additional file 1 — Nucleotide substitutions between each pair of Olea plastid genomes. [file 1471-2229-11-80-S1.DOC]

**Additional file S1.** **Nucleotide substitutions between each pair of *Olea* plastid genomes.**

| Accession | Manzanilla | Haut Atlas | Gué de Constantine | Imouzzer | Maui | Almihwit | Guangzhou |
| --- | --- | --- | --- | --- | --- | --- | --- |
| Haut Atlas | 44 |  |  |  |  |  |  |
| Gué de Constantine | 55 | 53 |  |  |  |  |  |
| Imouzzer | 43 | 43 | 50 |  |  |  |  |
| Maui | 99 | 102 | 103 | 97 |  |  |  |
| Almihwit | 101 | 102 | 106 | 98 | 75 |  |  |
| Guangzhou | 94 | 93 | 99 | 91 | 68 | 34 |  |
| *Olea woodiana* | 428 | 432 | 432 | 425 | 417 | 422 | 417 |
