# Supplementary material for: Genomic profiling of plastid DNA variation in the Mediterranean olive tree
Source: BMC Plant Biol. 2011 May 10;11:80. doi: 10.1186/1471-2229-11-80 (PMC3115843; doi:10.1186/1471-2229-11-80)
Supplement: Additional file 4 — PCR amplification and sequencing primers (5'->3') used to amplify and sequence the complete olive plastid genome. [file 1471-2229-11-80-S4.DOC]

**Additional file S4. PCR amplification and sequencing primers (5’–>3’) used to amplify and sequence the complete olive plastid genome.** The PCR primer annealing temperature (T_a_) and the amplicon size are given.

| Primer  pair no | Forward primer | Reverse primer | T_a_ (ºC) | Fragment size (bp) * | Nested sequencing primer |
| --- | --- | --- | --- | --- | --- |
| 1 (0A) | TTGGAGATACCATTGTTTCTGG | GGTCGAAATTCAACCTTTTGTC | 50 | 1371 | - |
| 2 (1A) | TGGGCGAACGACGGGAATTG | TAGTTCCGGGTTCGAATCCC | 53 | 1852 | F2. CACTCACGACCCATGTAACAA  R2. TTGTTATATGGGTCGTGAGTG  R3. GCTTCTATCGAAGTTCCATC |
| 3 (2A) | TCTCTCTAAAATTGCAGTCATG | CAACACGACTTTTTATATCCAC | 47 | 2039 | F2. TCGATATGACAGTGGGTTGC  F3. TTCTTCCAATAATTCCGAGCC |
| 4 (3A) | GGATACAAATTCTTGTTGCGCC | CTATATTTCCTTGAAAAGGGTGC | 50 | 2019 | F2. GAAGCCATAGGGCCCTATCC |
| 5 (4A) | TTCGTTTGATTAGGTCGAAGTTC | CTATGTACAACTGAACCAATGAC | 50 | 1212 | F2. GCTCAAATTGGATCCTTTCC |
| 6 (5A) | ACCACATCGTTTCAAACGAAG | TGGTAAGGCAACGGGTTTTG | 47 | 1742 | R2. AATAGGGGAGTGGATAGGAG |
| 7 (5Ab) | ATCTGTCCTTCAAGTCGCAC | GGGGTGATTTATCCTATCCC | 50 | 875 | - |
| 8 (6A) | GGGATCCCAAACCTTTTTCAC | GGAGGTTCGAATCCTTCCGT | 50 | 1030 | - |
| 9 (7A) | AATGGATATGGCTCTGGGAC | TTCACGTCCAGGATTACGTC | 50 | 1125 | - |
| 10 | *trnS-trnG* ; see Besnard *et al.* (2009) | | - | 1146 | - |
| 11 (8Ab) | ATAGTTAAAGGGTCTTTCGGTC | TCCTATCCATTAGACAATGGAC | 50 | 892 | - |
| 12 (8A) | AGCCTTCCAAGCTAACGATG | AATGTTGGTGTTGTATTAATGGG | 47 | 1711 | - |
| 13 (9A) | AACACGACCCAAATAGGCCTC | TTCGTTTCTTTGGGCCACTGG | 50 | 1624 | F2. CAGTCCCACATTATTGGTTCC  R2. TGGTTCGGGAAGGGATTATG  R3. TGGTAACCATTCGAGCCGAC |
| 14 (10A) | CCCTTTCCAAAAAAGATCAATAC | GTTGTTCTTGTTTCTTTAGTACC | 47 | 1860 | R2. GATAAAGAAAGTGGAAGGGAG |
| 15 (11A) | ATAAGCCGCGGCTAAAGTTG | TTTGATGCAGCAAGTAGGGG | 53 | 1464 | - |
| 16 (12A) | TTAACATAATGACACCGGGCC | TAGCCAAAAATGCACCACACC | 50 | 1430 | F2. TCTTCTACCGTAGATTGGCC  R2. GCAGTCATTTGATTTGGTAATC |
| 17 (13A) | GTGGCCAAATAAGGCTTAGC | TTCCCGAGGAAGTGCATATC | 53 | 1333 | - |
| 18 (14A) | CGTCGCTATGTCGAGATATC | TTATGGTCGGAGTCCTACTC | 53 | 1316 | F2. TCTCCACTCGGACAAACCCG  F3. TTCTTGAATCGATTGGAGTGG  F4. GTGTACGTGTCGGATGAACC |
| 19 (15A) | AGTACCTCCCGTGAATACTC | AGATAGTCCTTTGTGGCTCC | 53 | 1460 | - |
| 20 (16A) | AATATGACCAACAGTGGTTCGG | GCAATCGAGAAATCTCTACTGG | 50 | 1415 | - |
| 21 (17A) | GCCAATTCCATGCGTCTAAC | CTGAGGATGCTCCAGAATCT | 50 | 1655 | R2. CGGTATAAACATCAACAGCTACG |
| 22 (18A) | AGGATTTTAGTGGCCCAAGC | TCAAAAACCCCAGGTTCAGC | 50 | 1460 | - |
| 23 (19A) | GAATTGTAACCTTCCCATGGC | GGGGAGGAAGATCAGAATTAG | 50 | 1540 | - |
| 24 (20A) | GATTCGAACCCATAGCTGATG | GGAAAAAGGATTTGCAGTCCC | 50 | 1799 | F2. GTCCATTCTTCATCGAATTAGCG |
| 25 (21A) | TAGCATTGTGGATTTTCAGATAC | ATTTCGAAAAGGAAGAGTAAACC | 50 | 1684 | - |

**Additional file S4, continued**

| Primer  pair no | | Forward primer | Reverse primer | T_a_ (ºC) | Fragment size (bp) * | Nested sequencing primer |
| --- | --- | --- | --- | --- | --- | --- |
| 26 (1B) | TGGGGAAGAAGTGGACTCTA | | GCCAATCTACCATGAGATGG | 50 | 2118 | F2. GTGGTCCTCCTGTCAATAGT  F3. TTTACTTCGCAATAACTCGGG  R2. GATTCAAGATCGTCGAGAGG  R3. AGGAAGAGTAAACCTCACCG |
| 27 | | *trnD-trnT* spacer ; see Besnard (2008) | | - | 1064 | - |
| 28 (3B) | | GATGATTCAAGAATCAGCCAC | ACCAGCCTACAAAAACGAAAC | 47 | 1607 | F2. CCAAGTCACAAGATAAGAGCC |
| 29 (4B) | | TTGGTAAATTTACCAAAGACG | TATATCTTCTAAATCGTCCAC | 45 | 1695 | R2. GGTTTTCATGAGGCTGATCTTG  R3. GCACCATGAATAGCGCATAGC |
| 30 (5B) | | TTTTCTTCTAGTATTCAAGGCTC | ATACCCACCAGAAAGACTAAC | 47 | 1745 | F2. TCGTAGGTCATTTGTGGCAC |
| 31 (6Ba) | | CTCTTGTTTTCCAATTGGCTGT | CGACGTTGTTTTTCGTCCGG | 55 | 1066 | - |
| 32 (6Bb) | | GCAACAAACACGCATGAACC | CATCTTGTTTGGGCTACTGG | 50 | 645 | - |
| 33 (7B) | | CAAATTGGCCAAAGGTGTGCG | TCCGGTGTTTATCAGTGGTGG | 47 | 1720 | - |
| 34 (8B) | | CCTGCTATTAAGGATATGGCAG | TATCACATCTCAACTGGGCATG | 50 | 1433 | - |
| 35 (9B) | | CCATTGAGCAAAAACGGGTTG | CTCTTTGTATGTGTTGTCCGG | 50 | 1485 | - |
| 36 (10Ba) | | TTTTACGGGATCCCTATCCACC | CCGGGATGTCTCTTAGTAATGG | 50 | 474 | - |
| 37 (10Bb) | | TATGACATAGCCATGAGGCGC | GGGAGATTCTGAAATTGCGG | 50 | 684 | - |
| 38 (13B) | | GGAGTAAGCGCTATAGCCTGT | CGTTCGTACTTTGGATACCCG | 50 | 1405 | - |
| 39 (14B) | | CAACGCCTCCTAATTTCCAGG | AAGAGAGGGATTCGAACCCTC | 50 | 1444 | F2. CCCATAATAAAGAACCCCTAGG |
| 40 (15B) | | TAGGAGAGATGGCCGAGTGG | ATTCTATAGACACTATAGTGTAGTG | 50 | 1466 | F2. AACTAATTGGCGGGCTGCGG  R2. AGCCAAGGGGTCAACAGGTC |
| 41 | | *trnT-trnL* spacer ; see Besnard *et al.* (2009) | | - | 735 | - |
| 40 (1C) | | ATGACAATGAAATCCTAATCTC | GAATCTCTTATGATAATCATCC | 45 | 1808 | - |
| 41 | | *trnL-trnF* spacer ; see Besnard *et al.* (2009) | | - | 427 | - |
| 42 (2C) | | CTAGAACCGAAAGTACAATCC | TATCCATGGGCAATGAGTTTC | 50 | 1506 | - |
| 43 (3CaI) | | TTCAGGAGCTAAGACCATTCC | GGATTCCAGTCAAGTCAGTCG | 47 | 551 | - |
| 44 (3CaII) | | CGGATGGGTCGATTCGAATTG | AACACAAAGGGGATGGTCTTC | 53 | 571 | - |
| 45 (3Cb) | | GGAATAGAATCCGGAGTTTGTC | GTTTTACCGAGAAGGTCTACG | 50 | 736 | - |
| 46 (4Ca) | | GCTTTAAATGATTCCCGTGAG | TGGCGTATTACCAAATCATGC | 50 | 1554 | - |
| 47 (4Cb) | | TCACTACCCTTTTCCGCATCG | CCTACTACTTCTGGTTCTGGG | 50 | 1738 | F2. AAAATTCGTCCCAGAGTCGC |
| 48 (5C) | | CCGATGATTTGGACGATACG | CACCAGACATACGTAACGCT | 50 | 1759 | - |
| 49 (6C) | | CTTCACATTCACCGTGCAATG | TCCATAGAGTTTTCTTGCCCC | 50 | 1256 | - |
| 50 (7C) | | CGAAGACATAGGAGAAACCAC | CAAACTTCCTTCTTGCATGCG | 50 | 1384 | - |
| 51 (8CA) | | TAGTGGGGGAGAAAATCACC | TTAGAATGCGACCCAACAGG | 50 | 814 | - |
| 52 (8CB) | | TTTCTCTATCTCCCGAAAATCC | GGATGAGATTGGGTCCCATC | 55 | 525 | - |
| 53 (11B/9C) | | CCTAGTATTTCCGGCAATTGC | GGAACTAATAAAGAGACCCGC | 50 | 743 | - |

**Additional file S4, continued**

| Primer  pair no | Forward primer | Reverse primer | T_a_ (ºC) | Fragment size (bp) * | Nested sequencing primer |
| --- | --- | --- | --- | --- | --- |
| 54 (10C) | GTATGAGTTGGCGATCAGAAG | CCGAATGAACGAAAGAAGCAC | 50 | 1180 | - |
| 55 (-1D) | TCTTTCCCTCAATTATTCTATTC | TTGTTTCAGTTGCTTATCATAAG | 47 | 1606 | R2. GGAAAAGTGGAAACAAGACCAG |
| 56 (12C) | TAGCAGAATCGTGGATAGGG | TTGCTTACTATACCCGCTGC | 50 | 725 | - |
| 57 (13C) | AAGGGGTCAGATTTATCCCG | GTCTGTAGCATGACCACTTG | 50 | 1532 | F2. GAATTCCTTGTACGGCATTCC |
| 58 (1D) | GAAAATACCTATTAAACCAATCAC | GAGCTGAAGTTATAGTTAAAGC | 47 | 1985 | F2. TAACCCCCAGTAGAGACTGG  F3. CTTCCAGACATGCTCAGCTC  R2. TGGGGTCAATATCAGCAATGC |
| 59 (2Da) | CGTTTTATATGCGCCTGTCAC | GGATGTGGAAAACAAGACAGG | 47 | 1065 | - |
| 60 (2Db) | TTTGAACCCGTGACATTTTG | TTCTCAATTCTAATCGATTAGG | 47 | 1181 | F2. CTAGTTATTGACATGGGAAG |
| 61 (3D) | GTTCATGGCTAAGGGGAAAGA | GGAAGAAAGAACCCCATTCAC | 53 | 1888 | F2. CGAGTGATCCACAAACGACG  R2. GGATATATAGCTCGGAGACG |
| 62 (4D) | GTTCTTGACAGAGTGGGAGCT | CGTCAAAAGGTGCATGTACGG | 53 | 1763 | F2. TAAGCTTGGGCTTCTGTTGC |
| 63 (5D) | GATCTCGCTATCAACTTCTTGG | TCCAAAGATCTTGGGCAAATCC | 53 | 1810 | F2. GGGGAAATCCCATATGACCC  R2. TGTGTCCTTCCTTATTCCTC |
| 64 (6D) | GCACATATTGTGTTTTCGGG | GAAATACCGCAAATAGAGCC | 47 | 1984 | F2. AGTTAGTGCTGGGCTAGCAG  R2. CTGGGTACGCCTTATATACC |
| 65 (7D) | AGACGAACTACTGTAGGGAG | ATACTGGATCTTACGGAGCC | 47 | 2042 | R2. CTGTAACGGTCGGACGATAG  R3. GAAAGCTCTTCTTTGTGGCG |
| 66 (8D) | GATACAGAAGATAGGCTGGTTC | GGTATTCAAGAATCGGTCCATG | 50 | 2071 | F2. ATTACGGAGAACCCGCATGG  R2. ACAGTCCGGAAGATCTCATG |
| 67 (9D) | ACACAAATAGACGCGTCACAAG | GATCCGAAACTTCTTCCTATTTC | 50 | 1462 | F2. CATCTGTAACCGTGACAATGG |
| 68 (10D) | TTTCATTCCAGGTAAAGCCTCC | ATGGAACCTGTGCATGCAAAAG | 50 | 1995 | R2. CCTGAATATTGGGTAGCTG  R3. GAATTTTTGGTGCGATCCCC |
| 69 (11D) | GCCATCCCGACTAGTGAATC | GAGGAAGTTGTGGCACGTTC | 57 | 2164 | F2. TTCATACTATGGGGCTGGTC  F3. ACCCAAGACCCCATAGATAG  R2. GTCTTGGGTATCAAAATTTGG |
| 70 (12D) | TTGTGGGTATAATGGTAGATGC | = 1-For | 50 | 1363 | - |
| 71 (13D) | GATCGATTTCTAGGTTTCGTCG | AGTAGGATACTCCAAATTCGGG | 50 | 1276 | - |
| 72 (14Da) | CATGGACCCAATTCAATTCAGTG | TGCCTCCATTATGTTGTTGCTAG | 50 | 2051 | - |
| 73 (14Db) | GACCGCTATTCGAAATCTTAGTG | ATGTTCCTGGAAATTCTTGCTCC | 50 | 1703 | - |
| 74 (15D) | GGAATTCAAAGGGATCAAATAGG | ATTTAGATGTTTGTACCCTGTCG | 50 | 1470 | - |
| 75 (16D) | ATGCGTTGAGAAAGGGCAGATG | CCAAATAGGATCGTCCAGTTCC | 53 | 1289 | - |
| 76 (17D) | GAAAACCCTTTTCGCTCCGCT | GGAGGGTCCCAAATGAATTGG | 53 | 1654 | R2. TGTTGCGGGTCAAGGGCTCC |
| 77 (18D) | GATTCGGAGTTCTTGCAGAG | AATAGGCGTAATCGGACCTG | 50 | 1535 | F2. CATGGAACAATATGTTACTGCTG |
| 78 (19D) | GATGGCCAATCAAACCTATTTC | GCAAGTTCGTTATTACGGGTAG | 50 | 1612 | R2. GCTTCAGCTTCAGCCACTCG |

**Additional file S4, end**

| Primer  pair no | Forward primer | Reverse primer | T_a_ (ºC) | Fragment size (bp) * | Nested sequencing primer |
| --- | --- | --- | --- | --- | --- |
| 79 (20D) | TAGGGTGATCCTTTTGTCGAC | CCCATTGAAATAGGATCCACAC | 50 | 1605 | F2. ACTGAAACATTCTGGAGCTAC |
| 80 (21D) | CCCGGACGTTTTCGGGATGC | CTAACCCGTCAATTTGGGATCC | 53 | 1883 | F2. TTGACACGGACAAAGTCAGGG |
| 81 (22Da) | TATCCCATAGGTACAGTGTTTG | AATTGTCAACTGCCCCTATCG | 50 | 885 | - |
| 82 (22Db) | AGGAATTTGTCCATTTTTCGGG | CCAGGATCAAACTCTCCATGA | 50 | 604 | - |
| 83 (23D) | CGCATGGATACAAGTTATGCC | CTTGTTACGACTTCACTCCAG | 50 | 1544 | - |
| 84 (24D) | TTGTACACACCGCCCGTCAC | TGAGCCACCGGTTCAGGTAC | 53 | 1653 | R2. TGGTACGATCCCTCCGTCAC |
| 85 (25D) | AGCTCCGCTCTTGCAATTGGG | TCCCACTGCTTGGGAGCTTAC | 53 | 1560 | F2. AAGTTGTAAGCTGTGTTCGGG |
| 86 (26D) | ACTCCTGGGTGACCGATAGC | GTCAGCCCCCATACATGGTC | 53 | 1398 | - |
| 87 (27D) | CCCGTAACTTCGGGAGAAGG | CTCTACGCCTAGGACACCAG | 53 | 1570 | - |
| 88 (28D) | TGGAAGGGCAGAGGCCTTTG | GATAGACTAGAAACGACATCTG | 50 | 1440 | F2. TAACTCTTCCTCAGCCAGGC |
| 89 (29D/14E) | TGCTCTATTTCATTATATTCCATCC | GCATGTGTCAAAAAAATATTGTGAC | 50 | 1420 | - |
| 90 (OE) | ACTTTCGATTAACAGAGACAGG | TTGATCGACGAGTAATTGATGG | 50 | 272 | - |
| 91 (1E) | CACCCCCTACATATTTGATACC | ATAGGGTTTTGGTTCACACGAC | 50 | 1655 | - |
| 92 (2E) | AATCCCCTACACGATTAGTTAC | TTAGAATTCCCGGTAGAAAGAG | 50 | 1169 | - |
| 93 (3E) | TGGCAGTTCCAAAAAAACGTAC | ATCCACATAAAAGGGCTGCATA | 50 | 1619 | R2. GGTTCATTATTTTAGTTTAGGGCC |
| 94 (4E) | CAATCCGAGTGGTTAATAATGC | ACATACATGGTTACCAGATACC | 50 | 1644 | R2. TTGATTCTGGACCGCGAGAG |
| 95 (5E) | TTGCGCCTACTATGATCAACC | TGGTTAGAGCCCTTATGTGTC | 50 | 1696 | F2. TTGGTAGCTGAGTACAGACG  R2. CCTCTCTCATAAACCAATCCG |
| 96 (6E) | GCTTCAGCGGCTGCAATAGC | GGTAATTGCGTTGAGTATTGTCC | 50 | 1467 | - |
| 97 (7E) | TGATTATAATTCAATTCGTGACG | TCGACTATGATTATCTAACAGTT | 50 | 1039 | - |
| 98 (8Ea) | ATAAACCCTATAGGTTGACGCC | TATCTCTACGTGTGATTCGGTG | 50 | 1202 | - |
| 99 (8Eb) | ACTATCTGGTTTAGTTCATCAACC | GAAATCCGACGCTTTGATAGAG | 50 | 995 | - |
| 100 (9E) | AAACACTCTGATCTCCTATCAG | AGAGGGTCTGTTGAATTTCAAG | 50 | 1358 | - |
| 101 (10E) | ATTTTTCGCAAACCTCTCTGAG | GAATCGCTATTGGTTTGATACC | 47 | 638 | - |
| 102 (11E) | TAAGACAGGACGTGTGATGTG | CAAACCAACAAAAAGATATTGAAGAA | 50 | 919 | - |
| 103 (12E) | GCATTATATCCAAACTCATTTCTC | AAGGACTCATTTATTTCTAGATCG | 50 | 925 | - |
| 104 (13E) | TATTGATAGGGATACTCCTCAG | ATTCTAACCGTTGGAATAGACC | 50 | 3117 | F2. ACCATACGACGTTGATAAACTC  F3. CAGTATCAGGAAGAATGATACC  R2. ACTTGGTAAAGAATCAGGATCC  R3. GATACGCTATTCACAACAATCAG |

* Based on the *Olea europaea* subsp. *cuspidata* complete chloroplast genome, individual Maui 1 (GenBank accession FN650747).
