# Supplementary material for: Genomic profiling of plastid DNA variation in the Mediterranean olive tree
Source: BMC Plant Biol. 2011 May 10;11:80. doi: 10.1186/1471-2229-11-80 (PMC3115843; doi:10.1186/1471-2229-11-80)
Supplement: Additional file 5 — Characterised cultivars and their cpDNA haplotypes. [file 1471-2229-11-80-S5.DOC]

**Additional file S5.** **Characterised cultivars and their cpDNA haplotypes.**

| **Origin (boldface) & cultivar** | **Country** | **Origin** | **cpDNA haplotype** |
| --- | --- | --- | --- |
| **- Morocco** |  |  |  |
| Picholine Marocaine / Haouzia / Menara / Sigoise | Morocco | OWGB, INRAM & Field collect | E1-1 |
| Bouchouk | Morocco | INRAM | E1-1 |
| Chaouen, local type 1 | Morocco | Field collect | E2-4 |
| Chaouen, local type 2 | Morocco | Field collect | E2-4 |
| Chaouen, local type 3 | Morocco | Field collect | E2-4 |
| Hamrani | Morocco | INRAM | E1-1 |
| Chaouen, local type 4 | Morocco | Field collect | E2-1 |
| Ouazzane, local type 5 | Morocco | Field collect | E1-1 |
| Ouazzane, local type 6 | Morocco | INRAM | E3-2 |
| Ouazzane, local type 7 | Morocco | Field collect | E3-2 |
| Ouazzane, local type 9 | Morocco | Field collect | E3-2 |
| Bouchouk Laghlid | Morocco | INRAM | E1-1 |
| Taounate, local type 12 | Morocco | Field collect | E3-2 |
| Sefrou, local type 13 | Morocco | Field collect | E1-1 |
| Taounate, local type 14 | Morocco | Field collect | E3-2 |
| Taounate, local type 15 | Morocco | Field collect | L1-1 |
| Taounate, local type 16 | Morocco | Field collect | E1-1 |
| Sefrou, local type 17 | Morocco | Field collect | E3-2 |
| Sefrou, local type 18 | Morocco | Field collect | E3-2 |
| Meslala | Morocco | Field collect | E1-1 |
| Sefrou, local type 19 | Morocco | Field collect | E1-1 |
| Zarhoun, local type 20 | Morocco | Field collect | E1-1 |
| Zarhoun, local type 21 | Morocco | Field collect | E1-1 |
| Marrakech, local type 10 | Morocco | Menara garden | E1-1 |
| Marrakech, local type 26 | Morocco | Agdal garden | E1-1 |
| Marrakech, local type 28 | Morocco | Agdal garden | E1-1 |
| Marrakech, local type 29 | Morocco | Agdal garden | E1-1 |
| Marrakech, local type 30 | Morocco | Agdal garden | E1-1 |
| Marrakech, local type 32 | Morocco | Agdal garden | E1-1 |
| Marrakech, local type 33 | Morocco | Agdal garden | E1-1 |
| Marrakech, local type 34 | Morocco | Agdal garden | E1-1 |
| Marrakech, local type 35 | Morocco | Agdal garden | E1-1 |
| Marrakech, local type 36 | Morocco | Agdal garden | E1-1 |
| Marrakech, local type 38 | Morocco | Agdal garden | E1-1 |
| Marrakech, local type 40 | Morocco | Menara garden | E2-1 |
| Marrakech, local type 42 | Morocco | Menara garden | E1-1 |
| Marrakech, local type 43 | Morocco | Menara garden | E1-1 |
| Marrakech, local type 44 | Morocco | Menara garden | E1-1 |
| Marrakech, local type 45 | Morocco | Menara garden | E1-1 |
| Argana, local type 48 | Morocco | Field collect | E2-6 |
| Mentaga, local type 49 | Morocco | Field collect | E2-5 |
| Tamanar, local type 50 | Morocco | Field collect | E1-1 |
|  |  |  |  |

**Additional file S5, continued.**

| **Cultivars** | **Country** | **Origin** | **cpDNA haplotype** |
| --- | --- | --- | --- |
| **Algeria and Tunisia** |  |  |  |
| Chemlal de Kabylie | Algeria | OWGB | E3-2 |
| Taksrit | Algeria | Field collect | E1-1 |
| Chemlali | Tunisia | OWGB | E1-2 |
| Chetoui | Tunisia | OWGB | E1-2 |
| Gerboui | Tunisia | OWGB | E1-1 |
| Marsaline | Tunisia | OWGB | E1-1 |
| Meski | Tunisia | OWGB | E1-1 |
| Sfax, Local type 1 | Tunisia | Field collect | E1-1 |
| Sfax, Local type 2 | Tunisia | Field collect | E2-1 |
| Tunis, Local type 1 | Tunisia | Field collect | E1-1 |
| Tunis, Local type 2 | Tunisia | Field collect | E1-1 |
| Zalmati | Tunisia | OWGB | E1-1 |
| Zarazzi | Tunisia | INRAF | E3-2 |
|  |  |  |  |
| **- Iberian Peninsula** |  |  |  |
| Acebuchera | Spain | OWGB | E1-1 |
| Alfafara | Spain | OWGB | E1-1 |
| Aloreña de Iznalloz | Spain | OWGB | E1-1 |
| Arbequina | Spain | OWGB | E1-1 |
| Arbosana | Spain | OWGB | E1-1 |
| Argudell | Spain | OWGB | E1-1 |
| Asnal | Spain | OWGB | E1-1 |
| Bical | Spain | OWGB | E1-1 |
| Blanqueta | Spain | OWGB | E1-3 |
| Caballo | Spain | OWGB | E1-1 |
| Carrasqueño de Jumilla | Spain | OWGB | E1-1 |
| Castellana | Spain | OWGB | E1-1 |
| Changlot Real | Spain | OWGB | E1-1 |
| Cirujal | Spain | OWGB | E1-1 |
| Cornezuelo de Jaén | Spain | OWGB | E1-1 |
| Cornicabra | Spain | OWGB | E1-1 |
| Datilero | Spain | OWGB | E1-1 |
| Dulzal | Spain | OWGB | E1-1 |
| Empeltre | Spain | OWGB | E1-1 |
| Escarabajuelo de Posadas | Spain | OWGB | E1-1 |
| Farga | Spain | OWGB | E3-1 |
| Gordal de Granada | Spain | OWGB | E1-1 |
| Gordal Sevillana | Spain | OWGB | E1-2 |
| Habichuelero de Baena | Spain | OWGB | E1-1 |
| Hojiblanca | Spain | OWGB | E1-1 |
| Jaropo | Spain | OWGB | E1-1 |
| Lechín de Granada | Spain | OWGB | E1-1 |
| Lechín de Sevilla | Spain | OWGB | E2-3 |
| Llorón de Iznalloz | Spain | OWGB | E1-1 |
| Manzanilla Cacereña (Azeitera) | Spain | OWGB | E1-1 |
| Manzanilla de Almería | Spain | OWGB | E1-1 |
| Manzanilla de Sevilla | Spain | OWGB | E1-1 |
|  |  |  |  |

**Additional file S5, continued.**

| **Cultivars** | **Country** | **Origin** | **cpDNA haplotype** |
| --- | --- | --- | --- |
| Morisca | Spain | OWGB | E1-1 |
| Morrut | Spain | OWGB | E1-1 |
| Negrillo de La Carlota | Spain | OWGB | E1-1 |
| Nevadillo Blanco de Lucena | Spain | OWGB | E1-1 |
| Nevado Rizado | Spain | OWGB | E1-1 |
| Ocal | Spain | OWGB | E1-1 |
| Pico Limón | Spain | OWGB | E1-1 |
| Picual | Spain | OWGB | E1-1 |
| Picudo | Spain | OWGB | E1-1 |
| Sevillenca | Spain | OWGB | E1-1 |
| Verdial de Badajoz | Spain | OWGB | E1-1 |
| Verdial de Huévar | Spain | OWGB | E1-1 |
| Verdial de Vélez-Málaga | Spain | OWGB | E1-1 |
| Villalonga | Spain | OWGB | E1-3 |
| Cobrancosa | Portugal | OWGB | E1-1 |
| Galega Vulgar | Portugal | OWGB | E1-2 |
| Cordovil de Castelo Branco | Portugal | OWGB | E1-1 |
| Cordovil de Serpa | Portugal | OWGB | E1-1 |
|  |  |  |  |
| **- France (Mainland)** |  |  |  |
| Bouteillan | France | OWGB | E1-1 |
| Lucques | France | OWGB | E1-1 |
| Picholine | France | OWGB | E2-1 |
| Salonenque | France | OWGB | E1-1 |
| Tanche | France | OWGB | E1-1 |
| Cailletier | France | INRAF | E1-1 |
| Olivière | France | INRAF | E3-1 |
|  |  |  |  |
| **- Corsica and Sardinia** |  |  |  |
| Antonina | Corsica | Corte University | E3-2 |
| Capanacce | Corsica | Corte University | E1-1 |
| Curtinese | Corsica | Corte University | E1-1 |
| Migliaciaru | Corsica | Corte University | E1-1 |
| Oliese | Corsica | Corte University | E1-1 |
| Romana | Corsica | Corte University | E1-1 |
| Sabina | Corsica | Corte University | E2-1 |
| Zinzala | Corsica | Corte University | E2-2 |
| Bosana | Sardinia | CPFS | E1-1 |
| Cariasina | Sardinia | CPFS | E1-1 |
| Confetto | Sardinia | CPFS | E1-1 |
| Pizz’è Cuaddu (Giarraffa) | Sardinia | CPFS | E1-2 |
| Pizz’è Carrogna | Sardinia | CPFS | E1-1 |
| Sivigliana da Olio | Sardinia | CPFS | E2-1 |
|  |  |  |  |
| **- Italy (Mainland and Sicily)** |  |  |  |
| Biancolilla | Sicily | Field collect | E1-2 |
| Celeste, local type | Sicily | Field collect | E1-1 |
| Ascolana Tenera | Italy | OWGB | E1-1 |
| Bonifati, local type | Italy | Field collect | E2-1 |
|  |  |  |  |

**Additional file S5, continued.**

| **Cultivars** | **Country** | **Origin** | **cpDNA haplotype** |
| --- | --- | --- | --- |
| Belvedere Maritima, local type | Italy | Field collect | E1-1 |
| Carolea | Italy | OWGB | E1-2 |
| Coratina | Italy | OWGB | E1-1 |
| Dolce Agogia | Italy | OWGB | E1-1 |
| Frantoio | Italy | OWGB | E1-1 |
| Itrana | Italy | CPFS | E1-1 |
| Leccino | Italy | OWGB | E1-1 |
| Monte San’Angelo, local type | Italy | Field collect | E1-1 |
| Moraiolo | Italy | OWGB | E1-1 |
| Nocellara Bellica | Italy | OWGB | E1-1 |
|  |  |  |  |
| **- Bosnia and Albania** |  |  |  |
| Lastovka | Bosnia-Herzegovina | OWGB | E1-1 |
| Levantinka | Bosnia-Herzegovina | OWGB | E1-1 |
| Oblica | Bosnia-Herzegovina | OWGB | E1-1 |
| Buga | Bosnia-Herzegovina | OWGB | E1-1 |
| Crnica | Bosnia-Herzegovina | OWGB | E1-1 |
| Pulazeqin | Albania | OWGB | E1-1 |
| Ulliri i Bardhë i Tiranës | Albania | OWGB | E1-1 |
| Ulliri i Kuq | Albania | OWGB | E1-1 |
|  |  |  |  |
| **- Greece and Turkey** |  |  |  |
| Amygdalolia | Greece | INRAF | E1-2 |
| Kalamon | Greece | OWGB | E1-1 |
| Konservolia | Greece | OWGB | E1-1 |
| Koroneiki | Greece | OWGB | E1-1 |
| Megaritiki | Greece | OWGB | E2-2 |
| Matsoidis | Greece | OWGB | E1-1 |
| Mirtolia | Greece | OWGB | E1-1 |
| Ayvalik (Edremit Yaglik) | Turkey | OWGB | E1-1 |
| Belluti | Turkey | OWGB | E1-1 |
| Bodrum, local type 1 | Turkey | Field collect | E1-1 |
| Bodrum, local type 2 | Turkey | Field collect | E1-1 |
| Cakir (Valanolia) | Turkey | OWGB | E1-1 |
| Domat | Turkey | OWGB | E1-1 |
| Memecik | Turkey | OWGB | E1-1 |
| Trylia (Gemlik) | Turkey | OWGB | E1-1 |
| Uslu | Turkey | OWGB | E1-1 |
|  |  |  |  |
| **- Levantine region** |  |  |  |
| Barnea | Israel | OWGB | E1-1 |
| Merhavia | Irsael | OWGB | E1-1 |
| Nabali | Israel | OWGB | E1-1 |
| Mount Carmel, local type | Israel | Field collect | E1-1 |
| Beladi (Souri) | Lebanon | OWGB | E1-1 |
| Abbadi | Syria | OWGB | E1-1 |
| Kaesi | Syria | OWGB | E1-1 |
| Chalchali | Syria | OWGB | E1-1 |
|  |  |  |  |

**Additional file S5, end.**

| **Cultivars** | **Country** | **Origin** | **cpDNA haplotype** |
| --- | --- | --- | --- |
| Chami | Syria | OWGB | E1-1 |
| Zaity | Syria | OWGB | E1-2 |
| Palmyre, local type | Syria | Field collect | E1-2 |
| Paphos, local type 1 | Cyprus | Field collect | E1-1 |
| Paphos, local type 2 | Cyprus | Field collect | E1-1 |
| Paphos, local type 3 | Cyprus | Field collect | E1-1 |
|  |  |  |  |
| **- Egypt, Libya and Sudan** |  |  |  |
| Aggezi Shami | Egypt | OWGB | E1-1 |
| Hamed | Egypt | OWGB | E1-1 |
| Toffahi | Egypt | OWGB | E1-2 |
| Wardan | Egypt | OWGB | E1-1 |
| Kufra, local type | Libya | Kew herbarium (Newberry, sn – 1933) | E1-2 |
| Nuba Mts, local type | Sudan | Field collect | E1-1 |
|  |  |  |  |
| **- Introduced range** |  |  |  |
| Azapa (Arauco) | Chile | OWGB | E1-1 |
| Mission | South Africa | Field collect | E1-1 |

OWGB: Olive World Germplasm Bank (Córdoba, Spain).

INRAF: National Center of Agronomic Research in Montpellier, France.

CFPS: Consorzio Provinciale per la Frutticoltura di Sassari.

INRAM: National Center of Agronomic Research in Morocco.

Menara and Agdal gardens: Ancient olive gardens in Marrakech (Morocco), including a part of the Moroccan germplasm as indicated by Charafi et al. (2008).
